# Electron Nuclear Dynamics Simulations of Proton Cancer Therapy Reactions: Water Radiolysis and Proton- and Electron-Induced DNA Damage in Computational Prototypes

**DOI:** 10.3390/cancers10050136

**Published:** 2018-05-06

**Authors:** Erico S. Teixeira, Karthik Uppulury, Austin J. Privett, Christopher Stopera, Patrick M. McLaurin, Jorge A. Morales

**Affiliations:** 1 Department of Chemistry and Biochemistry, Texas Tech University, Lubbock, TX 79409, USA; teixeira.erico@gmail.com (E.S.T.); karthik.uppulury@gmail.com (K.U.); patrick.mclaurin@ttu.edu (P.M.M.); 2 Department of Chemistry and Biochemistry, Lipscomb University, Nashville, TN 37204, USA; austin.privett@lipscomb.edu; 3 Department of Chemistry and Industrial Hygiene, University of North Alabama, Florence, AL 35632, USA; cstopera@una.edu

**Keywords:** proton cancer therapy reactions, time-dependent non-adiabatic chemical dynamics, water radiolysis, proton-induced DNA damage, electron-induced DNA damage

## Abstract

Proton cancer therapy (PCT) utilizes high-energy proton projectiles to obliterate cancerous tumors with low damage to healthy tissues and without the side effects of X-ray therapy. The healing action of the protons results from their damage on cancerous cell DNA. Despite established clinical use, the chemical mechanisms of PCT reactions at the molecular level remain elusive. This situation prevents a rational design of PCT that can maximize its therapeutic power and minimize its side effects. The incomplete characterization of PCT reactions is partially due to the health risks associated with experimental/clinical techniques applied to human subjects. To overcome this situation, we are conducting time-dependent and non-adiabatic computer simulations of PCT reactions with the electron nuclear dynamics (END) method. Herein, we present a review of our previous and new END research on three fundamental types of PCT reactions: water radiolysis reactions, proton-induced DNA damage and electron-induced DNA damage. These studies are performed on the computational prototypes: proton + H_2_O clusters, proton + DNA/RNA bases and + cytosine nucleotide, and electron + cytosine nucleotide + H_2_O. These simulations provide chemical mechanisms and dynamical properties of the selected PCT reactions in comparison with available experimental and alternative computational results.

## 1. Introduction

Proton cancer therapy (PCT) employs high-energy H^+^ projectiles to obliterate cancerous tumors [1,2,3,4,5,6]. The therapeutic effect of PCT results from the H^+^ radiation damage on the DNA of cancerous cells [1,2,3,4,5,6]. That type of accumulated and unrepaired DNA damage leads to several anomalies in the cancerous cells that ultimately provoke their apoptosis [1,2,3,4,5,6]. The applied H^+^ radiation enters the patient’s body as collimated beams of H^+^ projectiles with an initial kinetic energy of 70–250 MeV [1,2,3,4,5,6]. The H*^+^* projectiles travelling through the body progressively lose their energy to the tissues; the degree of cell damage is directly proportional to the amount of energy deposited. The radiation energy loss vs. the body penetration is relatively small at shallow penetrations but finally presents a sharp maximum of energy deposition at a deep point [1,2,3,4,5,6]. That characteristic maximum, known as the Bragg peak, happens just before the H^+^ projectiles stop traveling in deep tissues. Since Bragg peaks can be focused on a cancerous region of the body, PCT is able to inflict maximum DNA damage on that region and minimum damage on the surrounding healthy tissues. This selective focusing on cancerous areas cannot be attained with traditional X-ray radiation. The latter presents a broader energy deposition maximum not far after its entry to the body and a gradual energy loss during its travel through the body.

**Figure 1:**
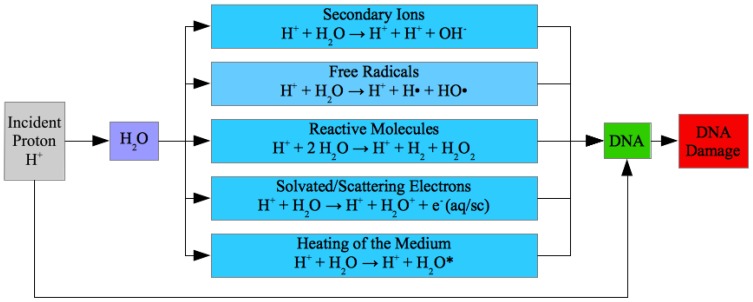
Scheme of the main chemical reactions and physical processes in PCT leading to DNA damage.

The biophysical basis of PCT is as follows [1,2,3,4,5,6]. Since water forms up to 70% of the human cell mass, the traveling H^+^ projectiles predominantly collide with H_2_O molecules. These collisions initiate water radiolysis reactions, i.e., a series of cascade reactions that produce various DNA-damaging species. Those species include (cf. Figure 1) (a) secondary ions (e.g., H^+^ + H_2_O → 2H^+^ *+* OH^−^) [3,4,6,7]; (b) free radicals (e.g., H^+^ + H_2_O → H^+^ + H·+ OH·) [3,4,6,7]; (c) reactive molecules (e.g., H^+^ + 2H_2_O → H^+^ + H_2_ + H_2_O_2_) [8]; (d) solvated/scattered electrons (e.g., H^+^ + H_2_O → H^+^ + H_2_O^+^ + *e*^−^(aq/sc)); and (e) localized heat in the medium [3,4,6,7]. These reactive species diffuse through the cytoplasm and reach the cell DNA causing various types of DNA damage: DNA bases’ fragmentations and deletions, DNA single-strand and double-strand breaks (SSBs and DSBs), and clustered DNA damage (CDD) that comprises several simple DNA lesions in close proximity to each other [3,4,5,6,9]. DSBs and CDD are particularly lethal to cells.

The biophysical picture of PCT is understood fragmentarily because many details of its mechanisms at the molecular level remain elusive [1,2,3,4,5,6,10,11]. This serious lacuna in the PCT knowledge precludes the emergence of a rational design of PCT that can help to complete its understanding, improve its therapeutic efficiency and mitigate its side effects. While experimental and clinical research strives to overcome this difficulty, computer simulations of the molecular-level processes underlying PCT can help to elucidate the fundamental mechanisms behind treatment techniques. This knowledge can be used to improve currently existing treatment protocols.

PCT involves numerous processes that span different space (l = 10^−10^–10^0^ m) and time (t = 10^−21^–10^5^ s) scales [3,4,6]. For example, water radiolysis reactions, DNA damage at the genome level [12] and clinical phenomena occur at the microscopic (10^−10^ ≤l≤ 10^−8^ m), mesoscopic (10^−8^ ≤l≤ 10^−3^ m) and macroscopic (10^−3^ ≤l≤ 10^0^ m) scales, respectively. A computational method for PCT should be appropriate for the scale of the processes to be simulated. For instance, microscopic water radiolysis reactions can be simulated with ab initio quantum-mechanics methods applied to computationally feasible prototypes (e.g., a large water cluster representing bulk cell water under radiolysis [7,13,14]). On the other hand, mesoscopic processes are only tractable with Monte Carlo (MC) models [12,15,16].

PCT research involving mesoscopic simulations has shown considerable development in recent years due to its relevance for dosimetry calculations [12,15,16]. In contrast, PCT research involving microscopic simulations is still under development due to the theoretical and computational challenges that quantum-mechanics methods experience when applied to large biomolecules. This lag in the quantum-mechanics methods is highly regrettable because microscopic and mesoscopic methods complement each other. For instance, microscopic simulations can inform mesoscopic simulations about the fundamental underlying processes that act at the molecular level. Furthermore, microscopic simulations can predict dynamical and kinetic properties difficult to measure in biological systems (e.g., water radiolysis reactions cross sections) that are the input data of mesoscopic simulations [12,15,16,17,18,19,20,21,22,23]. Despite the aforesaid challenges, several quantum methods were successfully applied to simulate PCT reactions, such as time-independent scattering methods [24,25], time-independent Hartree-Fock [26,27,28], Born-Oppenheimer molecular dynamics [29], and time-dependent non-adiabatic dynamics [7,13,14,30], inter alia (cf. Section 3 for more details about these methodologies).

In recent years, we have contributed to the simulation of various types of PCT reactions at the molecular level [7,13,14,30] with the electron nuclear dynamics (END) method [13,31,32]. END is a time-dependent, variational, on-the-fly, and non-adiabatic approach to simulate chemical reactions that can assume several versions with various levels of accuracy [13,31,32]. For instance, the simplest-level END (SLEND) version describes the nuclei in terms of classical mechanics and the electrons with a single-determinantal wavefunction [13,31,32]. Moreover, SLEND can be associated with time-dependent Kohn-Sham density-functional-theory (KSDFT) capabilities [33] to generate the SLEND/KSDFT method [13,34]; SLEND/KSDFT has a better description of electron correlation effects than SLEND [13,34]. Thanks to their structures, SLEND and SLEND/KSDFT have the proper balance between computational feasibility and accuracy to simulate various types of PCT reactions as demonstrated in our recent computational research on PCT [7,13,14,30].

The distinctive traits of our SLEND research on PCT arise from the special attributes of that method [13,31,32]. First and foremost, SLEND is capable of describing at once several of the processes that simultaneously occur during PCT: H^+^ projectile scattering, rovibrational excitations, fragmentation and rearrangement reactions, and electron-excitation and electron-transfer processes [7,13,14,30]. This avoids the less desirable use of miscellaneous methods that are tailor-made for specific types of scattering [24,25] and chemical [26,27,28,29] processes. On the computational side, and in addition to its feasibility, SLEND can calculate on-the-fly the forces acting among reactants during chemical reactions. This circumvents the calculation and construction of predetermined potential energy surfaces, a process that would be exceedingly cumbersome to conduct with the large biomolecules involved in PCT. Finally, but not least, SLEND is a time-dependent method and can therefore provide mechanistic details of a reaction at each time step during the conversion of reactants into products. This can be appreciated in the computer animations of PCT reactions presented in Section 3 that show the gradual transformation of reactants into products via intermediates.

In this contribution, we present to the cancer research community at large a review of our previous [7,13,14,30] and new END research on fundamental PCT reactions. This review portrays a panorama of the END capability to simulate various types of PCT reactions. The presented previous research includes simulations of water radiolysis reactions at collision energies of 1 and 100 keV performed on the computationally feasible prototypes: H^+^ + water clusters [7,14], and simulations of proton-induced DNA damage at 80 keV performed on the prototypes: H^+^ + DNA/RNA bases [30]. The presented new research includes additional simulations of proton-induced DNA damage at 1 keV but now performed on the more realistic and more computationally demanding H^+^ + cytosine nucleotide prototype. Furthermore, the new research also includes novel simulations of electron-induced DNA damage performed on the electron + cytosine nucleotide prototype. To the best of our knowledge, these time-dependent and non-adiabatic simulations of proton- and electron-induced DNA damage with the cytosine nucleotide are the first ever conducted with any method. Moreover, the simulations with the cytosine nucleotide involve the largest molecular system ever treated with END to date and constitute a significant step forward in the END research on PCT reactions.

## 2. Results

We performed all our PCT simulations with the SLEND [13,31,32] and SLEND/KSDFT [13,34] methods employing our END code PACE [13] (cf. Section 4 for details). To represent the electrons in the studied systems, SLEND utilized atomic basis sets of various sizes according to the computational cost of a given simulation [7,13,14,30] (STO-3G, 3–21G, 6–31G, and 6–31G++**) [35]. Similarly, SLEND/KSDFT utilized various combinations of KSDFT functionals and basis sets (B3LYP/STO-3G, BLYP/STO-3G, PBE/STO-3G, and B3LYP/3–21G) [30,33]. Water radiolysis reactions were simulated with the computational prototypes H^+^ + (H_2_O)_1–6_. Proton-induced DNA damage was simulated with the computational prototypes H^+^ + cytosine nucleotide and H^+^ + DNA/RNA bases. Electron-induced DNA damage was simulated with the computational prototypes electron + cytosine nucleotide (with H_2_O molecules for solvation in one case). The primary H^+^s traveling the Bragg peak area focused on a tumor have an energy of about 80–100 keV. Therefore, to reproduce primary Bragg-peak processes, the large studies of water radiolysis with H^+^ + (H_2_O)_1–6_ (25,020 simulations in total) [14] and of proton-induced DNA damage with H^+^ + DNA/RNA bases (806 simulations per each considered base) [30] are performed at 100 and 80 keV, respectively—the specific selection of 80 or 100 keV in each case (or any other energy in the 80–100 keV range) is determined by the availability of alternative results for comparison [14,30]. However, after passing through the Bragg peak area, the H^+^s continue travelling through the body at decreasing energies until stopping near the Bragg peak area focused on a tumor; these decelerated H^+^s continue causing PCT reactions near the cancerous area. Furthermore, the primary H^+^s at the Bragg peak area generate secondary, tertiary, etc., H^+^s (cf. Figure 1) that have energies below 80–100 keV; this secondary, tertiary, etc., species continue generating further water radiolysis reactions and DNA damage. Therefore, some simulations of water radiolysis reactions with H^+^ + (H_2_O)_2–3_ and of proton-induced DNA damage with H^+^ + cytosine nucleotide are performed at 1 keV; the latter energy is representative of decelerated primary H^+^s or of secondary, tertiary, etc. H^+^s. Simulations of the electron-induced DNA damage with electron + cytosine nucleotide are performed with low energy electrons (energy ≤ 20 eV) because these light particles are quickly decelerated by the medium. The simulations presented in the following sections rendered computer animations of the studied reactions that reveal the chemical mechanisms. In addition, these simulations predicted measurable dynamical properties (e.g., target-to-proton, bound-state-to-bound-state, one-electron-transfer total integral cross sections) that are compared with available experimental and alternative computational results. All the results are presented in full detail in the following sections along with in-depth discussions.

## 3. Discussion

### 3.1. Computer Simulation of Water Radiolysis Reactions in Water Clusters Prototypes

Water radiolysis reactions are at the core of the PCT process because they generate all the products leading to DNA damage [1,2,3,4,5,6]. Therefore, many computational studies of PCT concentrated on this type of PCT reaction. Water radiolysis reactions happen in bulk cell water. However, no current quantum-mechanics method can simulate bulk matter samples due to the prohibitive computational cost associated with those systems. Therefore, quantum-mechanics methods deal with computationally feasible prototypes that can capture bulk water effects. Surprisingly, most of those studies have utilized the smallest possible prototype: H^+^ + H_2_O [24,25,36,37,38], which ranks as the farthest from being a bulk water system. Despite that limitation, H^+^ + H_2_O studies could provide insightful details of water radiolysis processes [24,25,36,37,38], and even early SLEND studies were conducted on that simple system [36]. However, those studies could not completely capture the processes occurring in bulk water. Therefore, following a previous SLEND study on H^+^ +(H_2_O)_2_ [39], we have pioneered the SLEND simulation of PCT water radiolysis reactions using the water clusters prototypes H^+^ + (H_2_O)_1–6_ at the 1–100 keV collision energy range [7,13,14]. This progressive series of water clusters contains various isomers of each individual (H_2_O)*_n_* cluster and culminates in the prism isomer of (H_2_O)_6_. This isomer is considered as the “smallest drop” of water [40,41]—i.e., the minimum water portion that manifests the three-dimensional hydrogen-bond structure [41] and solubility properties [42,43] of bulk water. Therefore, our employed H^+^ + (H_2_O)_1–6_ series was devised to register the progression of water properties from the H_2_O molecule level to the earliest manifestations of bulk water.

**Figure 2:**
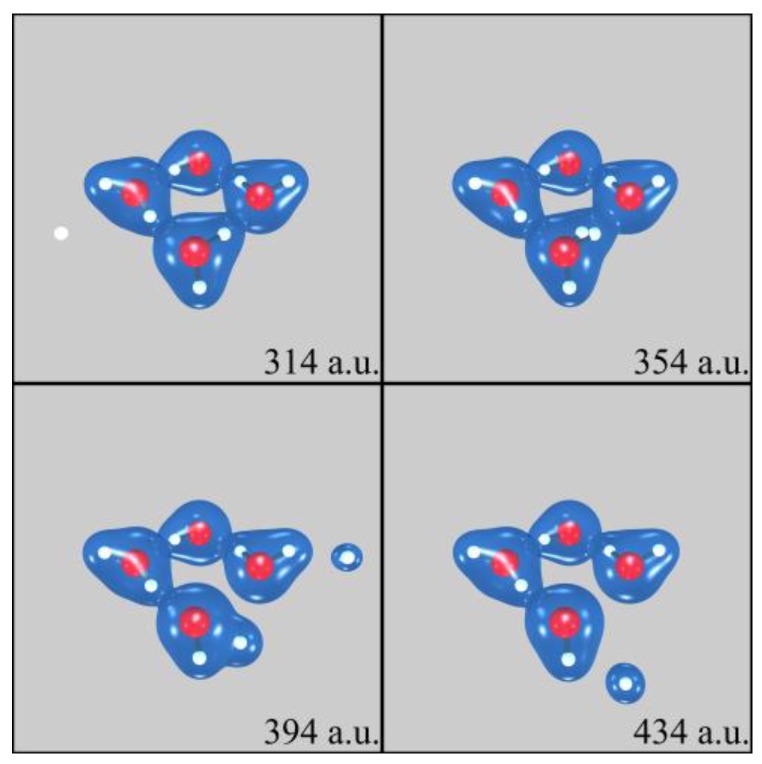
SLEND simulation of H^+^ + (H_2_O)_4_ at 1 keV at four different times shown in atomic units (a.u.). White and red spheres represent H and O atoms and blue clouds represent an electron density isosurface. The H^+^ projectile approaches (H_2_O)_4_ from the left (first panel), goes through it and takes some electron density (second and third panels), and forms the DNA-damaging H and OH radicals (third and fourth panels).

**Figure 3:**
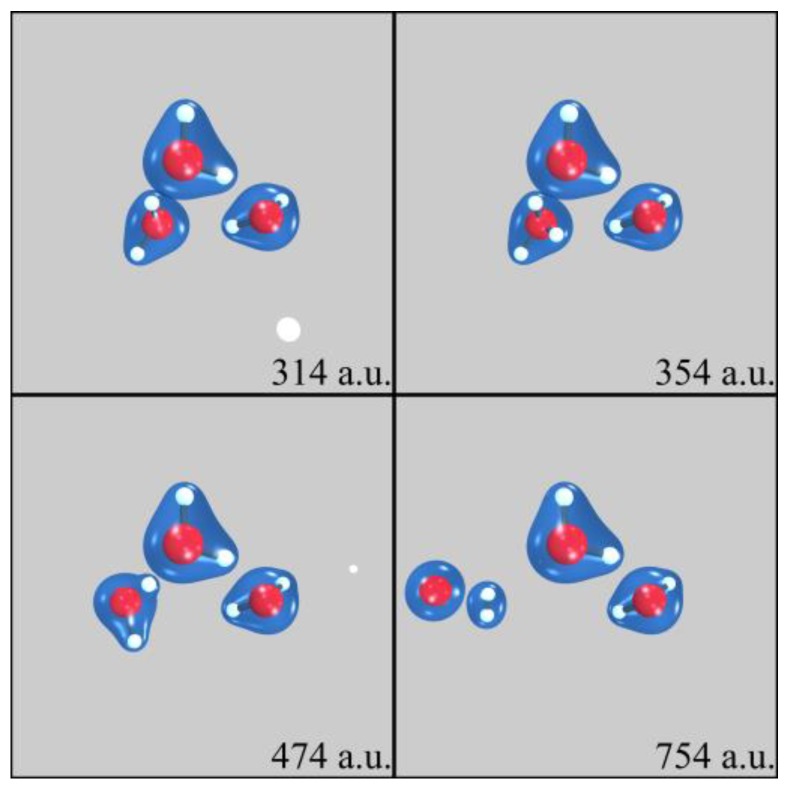
SLEND simulation of H^+^ + (H_2_O)_3_ at 1 keV at four different times shown in atomic units (a.u.). White and red spheres represent H and O atoms and blue clouds represent an electron density isosurface. The H^+^ projectile approaches (H_2_O)_3_ diagonally from the lower right corner (first panel), hits the lower left H_2_O molecule of the cluster (second panel), and bounces to the background (third panel). The collided H_2_O subsequently splits into an H_2_ molecule and a potentially DNA-damaging O radical (fourth panel).

One PCT aspect investigated in our SLEND simulations of H^+^ + (H_2_O)_1–6_ concerns the prediction of water radiolysis reactions that generate DNA-damaging species [7,13,14]. Figure 2 shows four sequential frames of a SLEND simulation of a selected collision of H^+^ + (H_2_O)_4_ at 1 keV calculated with the effective-core-potential/Stevens-Basch-Krauss-Jansien-Cundari (ECP/SBKJC) basis set [44]. The simulation times in Figure 2, in some subsequent figures, and throughout the text are given in atomic units (a.u., 1 a.u. = 2.41888425 × 10^−2^ femtoseconds). The shown cyclic (H_2_O)_4_ structure with near S_4_ symmetry is the most stable water tetramer [40,45]. In Figure 2, white and red spheres represent H and O atoms and blue clouds represent a selected electron density isosurface. Figure 2 shows that the H^+^ projectile approaches (H_2_O)_4_ from the left (first panel), goes through the cluster and takes some electron density (second and third panels), and forms the DNA-damaging H and OH radicals (third and fourth panels). The H radical is ejected from the cluster while the OH radical remains attached to it, partially solvated to the remaining three H_2_O molecules. Figure 3 shows four sequential frames of another SLEND simulation of a selected collision of H^+^ + (H_2_O)_3_ at 1 keV calculated with the 6–31G** basis set [35]. Figure 3 shows that the H^+^ projectile approaches (H_2_O)_3_ diagonally from the lower right corner (first panel), hits the lower left H_2_O molecule of the cluster (second panel), and bounces to the background (third panel). The collided H_2_O subsequently splits into an H_2_ molecule and a potentially DNA-damaging O radical (fourth panel). The reader can find in our Ref. [7] a complete analysis of the fragmentation products vs. the reactants initial conditions from 4602 and 1326 simulations of H^+^ + (H_2_O)_3_ and H^+^ + (H_2_O)_4_, respectively (Tables 2 and 3 and Figures 5 and 6 in that reference), and the corresponding fragmentation integral cross sections (ICSs; Section 3 therein). As all these results show, SLEND is capable of reproducing the main PCT water radiolysis reactions leading to DNA-damaging species.

**Figure 4:**
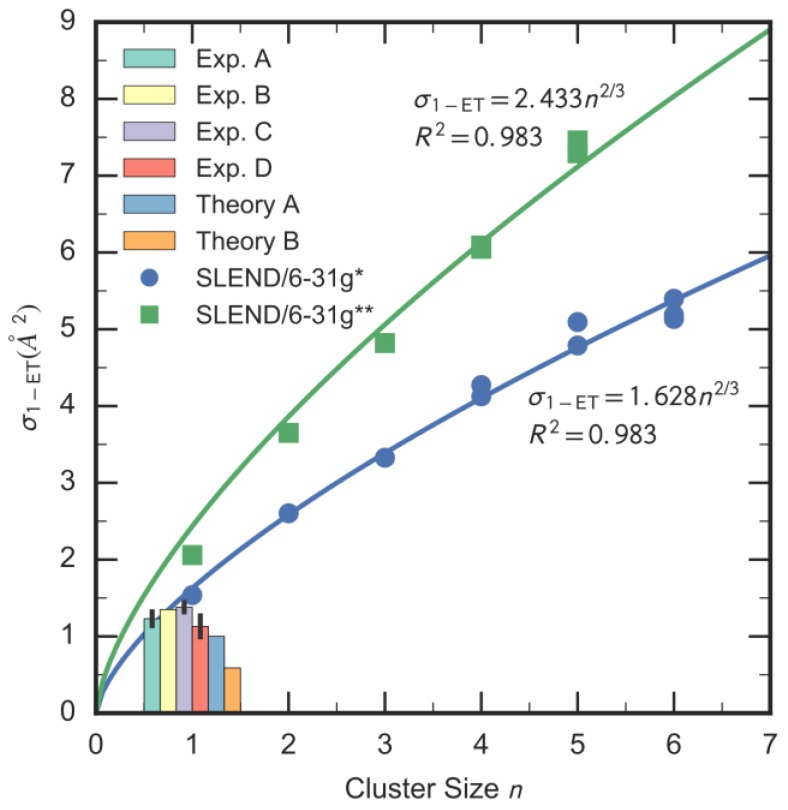
SLEND/6–31G* and /6–31G** cluster-to-proton bound-state-to-bound-state one-electron-transfer total integral cross sections, $\sigma_{1-ET}$, for H^+^ + (H_2_O)_1–6_ at collision energy = 100 keV vs. the water cluster size *n*. Current data are in comparison with available experimental and theoretical $\sigma_{1-ET}$ for *n* = 1 (Exp.: A [46], B [47], C [48] and D [49], Theory A: basis generator method (BGM) [37], Theory B: continuum distorted wave-eikonal initial state (CDW-EIS) approximation [25]). SLEND values are fit to the scaling formula $\sigma_{1-ET}\left(n\right)=cn^{2/3}$. Figure taken from our Ref. [14].

A better appraisal of the SLEND capability of simulating PCT water radiolysis reactions is given by the prediction of experimentally measured properties, e.g., the cluster-to-proton one-electron-transfer integral cross sections (ICSs), $\sigma_{1-ET}$, for H^+^ + (H_2_O)_1–6_ → H + (H_2_O)^+^_1–6_ at 100 keV [14]; the latter is an energy in the Bragg peak energy range [14]. These bound-state-to-bound-state electron-transfer reactions constitute by themselves an important type of water radiolysis reaction. The employed (H_2_O)_1–6_ series contained a total of 10 cluster isomers selected as follows [14]: the single H_2_O, (H_2_O)_2_, and (H_2_O)_3_ (cf. Figure 3) isomers, two (H_2_O)_4_ isomers [the most stable S_4_-symmetry-like isomer (cf. Figure 2) and a more asymmetric isomer], two (H_2_O)_5_ isomers (the most stable S_5_-symmetry-like isomer and a more asymmetric isomer), and three (H_2_O)_6_ isomers (the most stable prism isomer [40,41], the cage isomer, and a more asymmetric isomer; cf. [14,40] for further details about these structures). We performed these computationally demanding calculations with the 6–31G* [H^+^ + (H_2_O)_1–6_] and 6–31G** [H^+^ + (H_2_O)_1–5_] basis sets [35], an effort comprising a total of 25,020 simulations from various initial conditions of the reactants. Figure 4 shows the calculated SLEND $\sigma_{1-ET}$ for H^+^ + (H_2_O)_1–6_ at 100 keV vs. the cluster size n = 1–6 along with their experimental and theoretical counterparts only available for n = 1 (water monomer). The latter include data from four experiments denoted as Exp. A through D [46,47,48,49], respectively, and data from two alternative theories, denoted as Theory A: the basis generator method [37] (BGM) and Theory B: the continuum distorted wave-eikonal initial state (CDW-EIS) approximation [25]. In Figure 4**,** the average experimental ICSs, ${\bar{\sigma }}_{1-ET}^{Exp.}$, and its average relative error, $e_{Exp.}$, are 1.27 Å^2^ and ± 10.62%, respectively. The theoretical $\sigma_{1-ET}^{Theo.}$ and their average relative deviations ${\bar{\Delta }}_{Theo.}$ from the experimental values are: 1.54 Å^2^ and +21.8% for SLEND/6–31G*, 1.00 Å^2^ and +21.0% for BGM, and 0.589 Å^2^ and −53.4% for CDW-EIS. Only the BGM result is within the error bars of one experiment, Exp. D [49], with $\Delta_{Theo.}$ = 11.5%, but it lies on the lowest part of the error bar range. The SLEND/6–31G* result is very close to the result from Exp. C [48] with $\Delta_{Theo.}$ = 11.6% and not far from getting into the upper part of the error bar range. In absolute terms, the BGM and SLEND/6–31G* results are at the same level of accuracy and their agreement with the experimental data should be considered satisfactory given the difficulty to both measure and predict these electron-transfer processes. Deviations of the observed magnitude are not uncommon in measurements and predictions of similar complex processes (cf. Section 3.2 below for further examples). The CDW-EIS result compares less favorably with the experimental ones, being roughly half of its experimental counterparts (${\bar{\Delta }}_{Theo.}$ = −53.4%). The SLEND/6–31G** result also compares less favorably with the experimental ones, but, opposite to CDW-EIS, its value is roughly twice as much as the experimental one (${\bar{\Delta }}_{Theo.}$ = 63.0%). As discussed in our Ref. [14], this overestimation by SLEND/6–31G** arises from the contamination of the calculated bound-state-to-bound-state $\sigma_{1-ET}^{}$ with electron transfers to the continuum of unbound states. That overestimation can be remediated by projecting out those contributions; computational tools to perform that task are under development.

Figure 4 also shows the SLEND $\sigma_{1-ET}$ of H^+^ + (H_2_O)_2–6_. To the best of our knowledge, there are no experimental or theoretical data for comparison with the SLEND $\sigma_{1-ET}$ of H^+^ + (H_2_O)_2–6_; therefore, this investigation constitutes the first attempt to evaluate these $\sigma_{1-ET}$ and may prompt future experimental and theoretical studies to appraise these theoretical results. To analyze the incipient progression to the bulk water level, the SLEND $\sigma_{1-ET}$ in Figure 4 are fitted to the scaling formula $\sigma_{1-ET}\left(n\right)=cn^{2/3}$, where *c* is a coefficient and *n* is the cluster size. The derivation of this scaling formula is given in [14] (that derivation essentially relates the cross section areas with the effective external areas of the clusters growing with *n*). Figure 4 plots the $\sigma_{1-ET}\left(n\right)=cn^{2/3}$ curves and shows their coefficients c and correlation factors $R^{2}$. The SLEND/6–31G* and SLEND/6–31G** $\sigma_{1-ET}$ fit extremely well into those formulae, a fact suggesting that the SLEND results scale correctly to the bulk water level. A closer inspection of Figure 4 indicates that the SLEND $\sigma_{1-ET}$ for the various isomers appearing at *n* ≥ 4 do not significantly differ in their values; this implies that these $\sigma_{1-ET}$ values are rather unaffected by the isomers’ structures. In fact, this phenomenon permits fitting all the isomers data with a single $\sigma_{1-ET}\left(n\right)=cn^{2/3}$ formula instead of fitting different families of isomers (quasi-planar, bulky, etc. isomers) with different formulae. Moreover, we expected that the $\sigma_{1-ET}$ of the drop-like prism and cage (H_2_O)_6_ isomers would differ considerably from the $\sigma_{1-ET}$ of the rest of the quasi-planar/multiplanar isomers, a fact that would have prevented fitting drop-like and non-drop-like $\sigma_{1-ET}$ with a single formulae. Such a hypothetical fitting failure would manifest as a “phase transition” discontinuity from non-drop-like to drop-like $\sigma_{1-ET}$. However, no such “phase transition” is observed in the selected series of (H_2_O)_1–6_ isomers. Thus, unlike the case of structural and solubility properties [41,42,43], the prism and cage (H_2_O)_6_ isomers do not bring about any manifestation of water radiolysis processes in bulk water. In order to finally reach the bulk water level, we are currently simulating the H^+^ + (H_2_O)_7–22_ series, where bulk water properties are supposed to emerge in water clusters with two solvation shells, e.g., (H_2_O)*_n_* with *n* ≥ 17.

### 3.2. Computer Simulations of Proton-Induced DNA Damage in Nucleotide Prototypes

**Figure 5:**
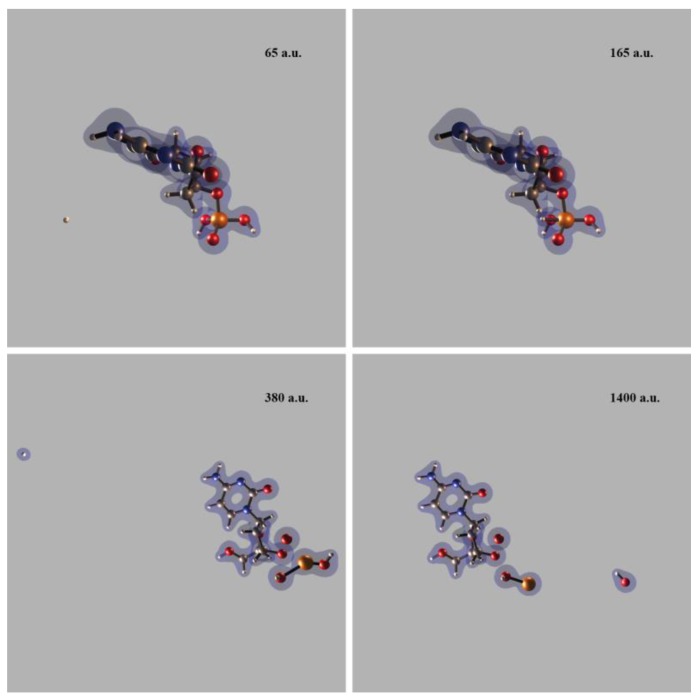
SLEND simulation of a proton-induced DNA SSB in H^+^ + excised cytosine nucleotide at 1 keV (frame times shown in atomic units (a.u.)). Colored spheres represent atoms (white = H, gray = C, red = O, blue = N, and orange = P) and the transparent clouds represent an electron density isosurface. The H^+^ projectile approaches the nucleotide from the left aiming at the P atom of the 3′ phospho-ester bond (first panel), hits that atom (second panel), breaks the P−O bond and bounces back to the far left (third panel); meanwhile, POH, OH, O moieties dissociate from the rest of the nucleotide structure (third and fourth panels). The camera’s point of view changes in the last two frames to facilitate the fragments’ visualization.

Protons are one of the most abundant harmful particles capable of damaging DNA in PCT. Protons attacking DNA molecules in deep tumor cells could be either decelerated primary protons or secondary protons generated by the water radiolysis reactions. For this type of DNA damage, there are no established hypotheses about its mechanisms aside from valid speculations about proton-induced DNA bases’ fragmentation and deletion, DNA sugar-phosphate lesions, DNA helix opening, DNA SSBs and DSBs [3,24]. Due to prohibitive computational cost, most quantum-mechanics computer simulations of this type of damage are based on the H^+^ + DNA base prototypes that can only capture the base part of proton-induced DNA damage [24,50]. While proton-induced DNA base damage is important in PCT, simulations restricted to that part of DNA miss relevant processes at the sugar-phosphate backbone, for instance DNA SSBs. Therefore, to overcome that limitation, we are currently conducting the first ever simulations of proton-induced DNA SSBs on the computational prototype H^+^ + excised cytosine nucleotide [26,27,28] at 1 keV of collision energy. Figure 5 shows four sequential frames of a SLEND simulation of that prototype conducted with the STO-3G basis set [35]. Therein, colored spheres represent atoms (white = H, gray = C, red = O, blue = N and orange = P) and the transparent clouds represent a selected electron density isosurface. In Figure 5, the H^+^ projectile approaches the nucleotide from the left aiming at the P atom of the 3′ C−O−P phospho-ester bond (first panel), hits that atom (second panel), breaks the P−O bond and bounces to the far left (third panel); meanwhile, POH, OH, O moieties dissociate from the rest of the nucleotide structure (third and fourth panels). Notice that the camera’s point of view changes in the last two frames to facilitate the fragments visualization. The net result of this process is a DNA SSB at the 3′ C−O−P phospho-ester bond and the formation of the DNA-damaging radicals POH, OH and O.

**Figure 6:**
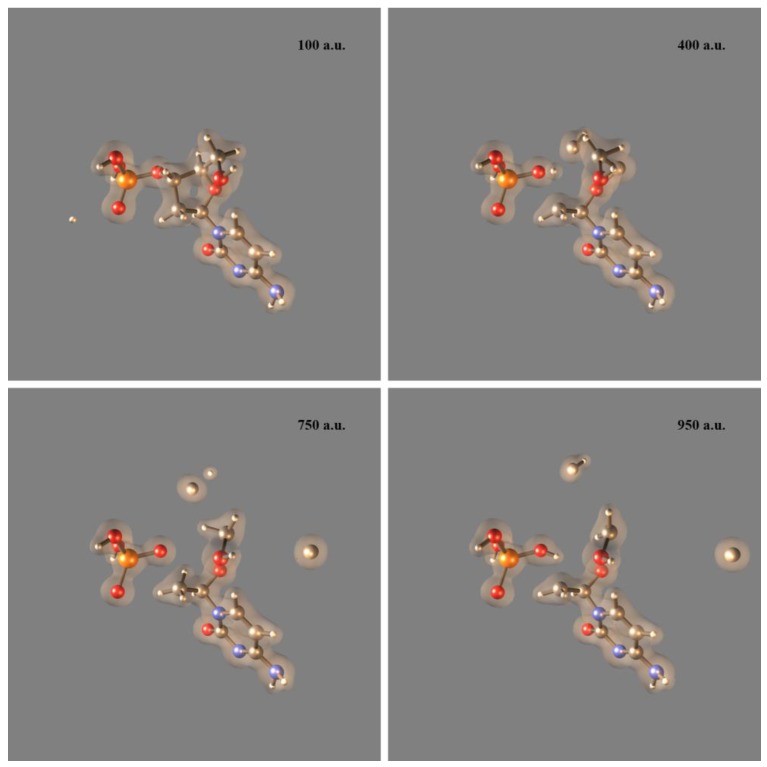
SLEND simulation of a proton-induced DNA SSB in H^+^ + excised cytosine nucleotide at 1 keV (frame times shown in atomic units (a.u.)). Colored spheres represent atoms (white = H, gray = C, red = O, blue = N, and orange = P) and the transparent clouds represent an electron density isosurface. The H^+^ projectile approaches the nucleotide from the left aiming at the C atom of the 3′ phospho-ester bond (first panel), hits that atom, breaks that bond and scatters away (second panel); meanwhile, the nucleotide breaks into CH_2_OH, H_3_PO_4,_ CH, and C moieties and the rest of its structure. During the collision, one H atom migrates from the CH_3_ group hanging from the damaged sugar to the detached H_2_PO_4_ group to form a H_3_PO_4_ molecule (third and the fourth panels).

Figure 6 shows four sequential frames of another SLEND simulation of the same prototype conducted again with the STO-3G basis set [35]. In this case, the H^+^ projectile approaches the nucleotide from the left aiming at the C atom of the 3′ C−O−P phospho-ester bond (first panel), hits that atom, breaks that bond and scatters away (second panel); meanwhile, the nucleotide breaks into CH_2_OH, H_3_PO_4_, CH and C moieties and the rest of its structure. Remarkably, during this collision, one H atom migrates from the CH_3_ group hanging from the damaged sugar to the detached H_2_PO_4_ group to form a H_3_PO_4_ molecule (third and the fourth panels). The net result of this process is another type of a DNA SSB at the 3′ C−O−P phospho-ester bond and the formation of a H_3_PO_4_ molecule and the DNA-damaging radicals CH_2_OH, CH, and C. These simulations of proton-induced DNA damage with a nucleotide prototype provide an interesting glimpse of this type of damage for the first time.

**Figure 7:**
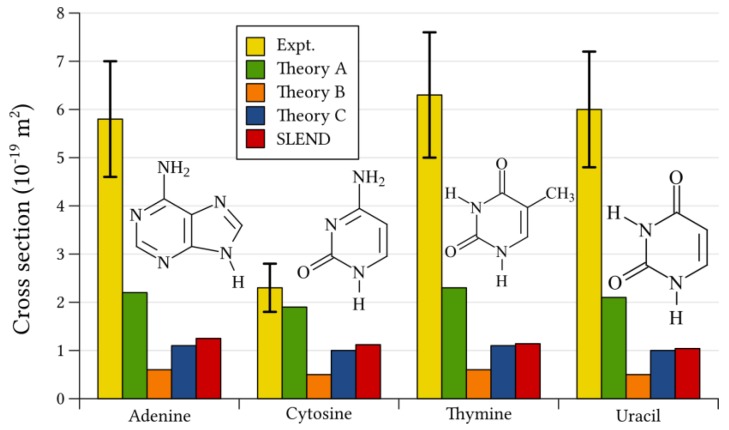
Base-to-proton**,** one-electron-transfer, total integral cross sections, $\sigma_{1-\mathrm{ET}}$, for H^+^ + DNA/RNA base → H + DNA/RNA base^+^ at 80 keV from experiment [51], from previous theories (Theory A, continuum distorted wave (CDW) approximation [24]; Theory B, continuum distorted wave-eikonal initial state (CDW-EIS) approximation [24]; and Theory C, classical trajectory Monte Carlo with classical-over-barrier (CTMC-COB) criteria approach [50]) and from SLEND with mixed basis sets: 6–31++G** for the projectile H atom and 6–31G for the rest of the atoms.

As with water radiolysis reactions, a better appraisal of the SLEND capability of simulating proton-induced DNA damage is given by the prediction of experimentally measured properties. However, unlike water radiolysis reactions, there are scarce experimental data for the present type of reaction due to the difficulty to prepare gas-phase nucleotide samples for H^+^-beam scattering experiments. To the best of our knowledge, there are no experimental data for H^+^ + nucleotide reactions. Fortunately, there is a pioneering experiment [51] on the one-electron-transfer reactions: H^+^ + DNA/RNA base → H + DNA/RNA base^+^ at 80 keV, where DNA/RNA base = adenine, cytosine, thymine, and uracil [51]. This experiment surveyed the base part of proton-induced DNA in PCT. Consequently, we performed simulations of H^+^ + DNA/RNA base at 80 keV with SLEND associated with several basis sets: STO-3G, 3–21G, 6–31G, and 6–31G/H^+^++** [30]. The latter is a mixed basis set combining 6–31G++** with diffuse (++) and polarization (**) functions [35] for the projectile H atom and 6–31G [35] for the rest of the atoms; the augmented basis set on the projectile atom improves the simulation of electron transfers to that atom. We also performed additional simulations with SLEND/KSDFT associated with several functionals and basis sets: B3LYP/STO-3G, BLYP/STO-3G, PBE/STO-3G, and B3LYP/3–21G [30,33]. This study comprises 806 simulations per each considered base and method (e.g., adenine with SLEND/STO-3G, adenine with SLEND/KSDFT/B3LYP/STO-3G, etc.). Figure 7 shows the one-electron-transfer total ICSs $\sigma_{1-\mathrm{ET}}$ for the aforesaid H^+^ + DNA/RNA base systems from the experiment [51], from the best of our SLEND simulations, SLEND/6–31G/H^+^++** [30], and from three alternative theories, namely, Theory A: the continuum distorted wave (CDW) approximation [24], Theory B: the continuum distorted wave-eikonal initial state (CDW-IES) approximation [24], and Theory C: the classical trajectory Monte Carlo with classical-over-barrier (CTMC-COB) [50] method. On average, the absolute and relative percentage errors in the experimental ICSs $\sigma_{1-\mathrm{ET}}^{\mathrm{Expt}.}$ are $\varepsilon^{Expt.}$= 1.050 × 10^−19^ m^2^ and $\varepsilon^{Expt.}\;100/\sigma_{1-\mathrm{ET}}^{\mathrm{Expt}.}$ = 20.8%, respectively. On the other hand, on average, the absolute deviation from the experimental value $\left|\sigma_{1-\mathrm{ET}}^{\mathrm{Expt}.}-\sigma_{1-\mathrm{ET}}^{\mathrm{Theory}}\right|$ (10^−19^ m^2^) and the relative percentage deviation from the experimental value $\left|\sigma_{1-\mathrm{ET}}^{\mathrm{Expt}.}-\sigma_{1-\mathrm{ET}}^{\mathrm{Theory}}\right|$$100/\sigma_{1-\mathrm{ET}}^{\mathrm{Expt}.}$ for the theoretical ICSs $\sigma_{1-\mathrm{ET}}^{\mathrm{Theory}}$ are: 2.98 and 52.0% (CDW), 4.55 and 87.5% (CDW-IES), 4.05 and 75.8% (CTMC-COB), 4.11 and 77.1% (SLEND), and 4.46 and 77.6% (SLEND/KSDFT), respectively. Again, like with water radiolysis reactions, experimental errors and theoretical deviations are considerable as one might expect in dynamical measurements and calculations of these complex systems. Therefore, the performances of all the considered theoretical methods in predicting the experimental $\sigma_{1-\mathrm{ET}}^{\mathrm{Expt}.}$ can be considered reasonable. Without exception, all the theoretical values from all the examined methods are smaller than their corresponding experimental values. With one exception, all the theoretical values lie outside the experimental error interval. The only exception is for H^+^ + cytosine with the CDW approximation, where $\sigma_{1-\mathrm{ET}}^{\mathrm{CDW}}$ = 1.9 < $\sigma_{1-\mathrm{ET}}^{\mathrm{Expt}.}$ = 2.3 but $\sigma_{1-\mathrm{ET}}^{\mathrm{CDW}}$ = 1.9 > $\sigma_{1-\mathrm{ET}}^{\mathrm{Expt}.}$ $-\varepsilon^{Expt.}$ = 2.3–0.5 = 1.8 (10^−19^ m^2^). Except for SLEND/KSDFT/STO-3G [30], all the examined theoretical methods predict ICSs $\sigma_{1-\mathrm{ET}}^{\mathrm{Theory}}$ that barely differ in value along the four considered DNA/RNA bases. This uniformity was also observed with the water clusters discussed in Section 3.1. This uniformity is also observed in the experimental $\sigma_{1-\mathrm{ET}}^{\mathrm{Expt}.}$ for adenine, thymine and uracil but not for cytosine, whose experimental ICS $\sigma_{1-\mathrm{ET}}^{\mathrm{Expt}.}$ is considerably lower than those of the remaining bases. Roughly speaking, all the highly different theoretical methods predict ICSs $\sigma_{1-\mathrm{ET}}^{\mathrm{Expt}.}$ of ≈0.5–2 (10^−19^ m^2^) for all the bases, while the experiment predicts values of ≈6 (10^−19^ m^2^) for all the bases except cytosine. This consistent and large discrepancy between theoretical and experimental results calls for a careful reexamination of all these results and methodologies. The present results establish the following ranking for the considered theoretical methods in their accuracy to predict the current experimental $\sigma_{1-\mathrm{ET}}^{\mathrm{Expt}.}$ [30]: CDW > CTMC-CO ≈ SLEND ≈ SLEND/KSDFT > CDW-EIS. In the case of SLEND/KSDFT, it is interesting to note that with the same basis sets, $\sigma_{1-\mathrm{ET}}^{\mathrm{Theory}}$ with the B3LYP, BLYP and PBE functionals are almost identical [30]. To correctly interpret the ranking of SLEND and SLEND/KSDFT, it is appropriate to remember that those methods are time-dependent, ab initio, and capable of describing several types of processes involving all particles (scattering, fragmentations, electron transfers, etc.). In contrast, the alternative theoretical methods are time-independent (CDW and CDW-EIS), rely on parametrizations with respect to experimental data to achieve accuracy (CTMC-CO), and are tailor-made for scattering processes involving a few active electrons (CDW and CDW-EIS).

### 3.3. Computer Simulations of Electron-Induced DNA Damage in Nucleotide Prototypes

Electrons generated in the PCT water radiolysis reactions produce considerable DNA damage during that therapy. In fact, electron-induced DNA damage is an important process not only in PCT but in any type of ionizing radiation therapy that generates electrons via water radiolysis (e.g., carbon ion and electron cancer therapies). Furthermore, electron-induced DNA damage plays an important role in oncogenesis processes that result from human exposure to electron-carrying radiation (e.g., beta radiation from radioactive materials and nuclear reactors and solar wind radiation; cf. Ref. [52] for a comprehensive review on dissociative electron attachment in physical, biological and astrophysical systems).

Experimental [53,54,55,56,57,58,59] and computational [26,27,28,60] research on electron-induced DNA damage has concentrated on the determination of the capture mechanisms and sites of the impinging electron (on different parts of the DNA: base, sugar or phosphate) and of the chemical reactions that arise from those captures (atom ablations from the base, base detachments from the sugar-phosphate backbone, DNA SSBs, etc.). There is experimental evidence that low-energy electrons with kinetic energy ≤20 eV inflict considerable damage on DNA [53,54,55,56,57,58,59]. Low-energy electrons abound in PCT because just after being generated, the very light electrons are considerably decelerated by their collisions with H_2_O molecules. Therefore, like other research groups, we will consider herein the role of low-energy electrons in PCT. From an extensive body of experimental [53,54,55,56,57,58,59] and computational data [26,27,28,60], the following picture of DNA damage by low-energy electrons emerge. At the high end of the low-energy electron range (≥10 eV), experiments show that the impinging electrons attach to low-energy $e^{-}$ orbitals of the DNA bases by forming core-excited resonant states: $\pi^{2}$ + $\pi^{2}$ → $\pi^{1}\pi^{*2}$ (i.e., the impinging electron excites a bound electron from a *π* orbital to end up together in a $\pi^{*}$ orbital) [28,53]. The formed $\pi^{*2}$ electrons can inflict damage on the capturing DNA bases or transfer to other parts of the DNA and produce SSBs and other damage there [28,53]. At the low end of the low-energy electron range (≤ 3 eV), experiments show that the impinging electrons attach to low-energy $\pi^{*}$ orbitals through shape-resonant states: $e^{-}$ + $\pi^{*}$ → $\pi^{*1}$ [54,55,56,57,58]. The specific site of the electron capturing $\pi^{*}$ orbital can be on the DNA bases, phosphates, or sugars (captures at the sugars have been the less studied so far). If the electron attaches to a π* orbital of a DNA base, it can produce in situ [26,27,28,54,55,56,57,58] cleavage of the base N−H amine bond and cleavage of the C−N bond between the base and the sugar (this prompts the base detachment from the DNA sugar-phosphate backbone); furthermore, the captured electron can transfer from the base through the sugar to a 3′ C−O $\sigma^{*}$ phospho-ester bond (or close ones, e.g., P−O) where the excess electron in that anti-bonding bond produces its cleavage. The last process is highly relevant for PCT because it constitutes a DNA SSB; it also involves a still unknown mechanism of intramolecular electron transfer to achieve a SSB. If the electron attaches to a π* orbital of a phosphate, it can transfer from there to produce a SSB on the contiguous 3′ or 5′ C−O $\sigma^{*}$ phospho-ester bonds (or close ones, e.g., P−O) of the sugar-phosphate backbone. The Simons group has studied electron-induced DNA damage from base and phosphate capturing sites via time-independent Hartree-Fock calculations on excised nucleotides (e.g., the excised cytosine nucleotide) [26,27,28]. Those calculations were not real time-dependent simulations of reactions but provided potential energy surfaces from which dynamical and kinetic processes can be inferred. This group determined that electrons with energies 2 eV attach to the DNA base π* lowest occupied molecular orbital (LUMO) and cause a 3′ C−O $\sigma^{*}$ phospho-ester SSB via an electron transfer through the sugar described above (according to these authors, this process predominates over the alternative N−H amine base cleavage and DNA base detachment also described above). On the other hand, electrons with energies > 2 eV attach to the phosphate P=O $\pi^{*}$ orbital and produce a 3′ or 5′ C−O $\sigma^{*}$ phospho-ester bond SSB. These calculations suggested the predominance of the SSB mechanism from base capture over that from phosphate capture and over other conceivable alternatives[28]. However, a more recent experiment [59] of low-energy electron irradiation on the gas-phase 2′-deoxycytidine 5′-monophosphate showed that SSBs from electron captures at the phosphate, sugar and base occur with relative contributions of 60%, 25%, and 15%, respectively. Additional computational studies of electron-induced DNA damage in nucleotides also studied solvation [26,27,28,29] and vibrational effects [26,27,28] on the corresponding reactions.

Undoubtedly, the discussed computational studies of electron-induced DNA damage have provided valuable insight into that process. However, they could not provide a complete time-dependent and non-adiabatic description of electron-induced type of DNA damage (those studies employed time-independent Hartree-Fock [26,27,28] and Born-Oppenheimer (ground-state) molecular dynamics [29] methods). Only time-dependent simulations can capture all the details of chemical reactions and only a non-adiabatic framework can describe the involved electron transfers. Fortunately, SLEND provides that convenient time-dependent and non-adiabatic picture. Thus, we are presently investigating with SLEND electron-induced DNA damage employing the same computational prototype of Refs. [26,27,28]: the excised cytosine nucleotide with three added H atoms to cap the severed bonds and neutralize the negative charge. The previously considered computational methods and the current SLEND version cannot describe the capture of the impinging electron via a shape resonance—we are developing a SLEND version based on plane-wave basis sets [61] to describe that actual capture, but those capabilities are not available presently. Therefore, like in previous studies [26,27,28], we start our simulations just after the electron has been captured, with the electron attachment achieved by the insertion of the electron in an unoccupied (virtual) orbital that acts as a metastable shape-resonant state.

**Figure 8:**
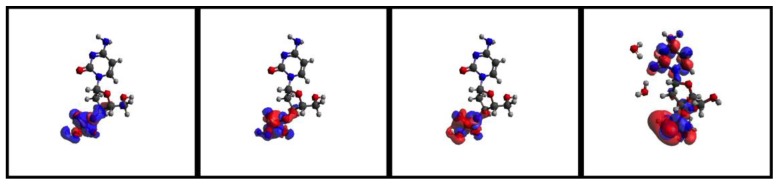
Some virtual orbitals of the excised cytosine nucleotide with high localization on the phosphate. For the dry nucleotide, LUMO + 2$\sigma^{*}$(first panel), LUMO + 3$\pi^{*}$ (second panel), and LUMO + 4$\pi^{*}$ (third panel). For the nucleotide + 4 H_2_O, LUMO + 4$\pi^{*}$ (fourth panel).

**Figure 9:**
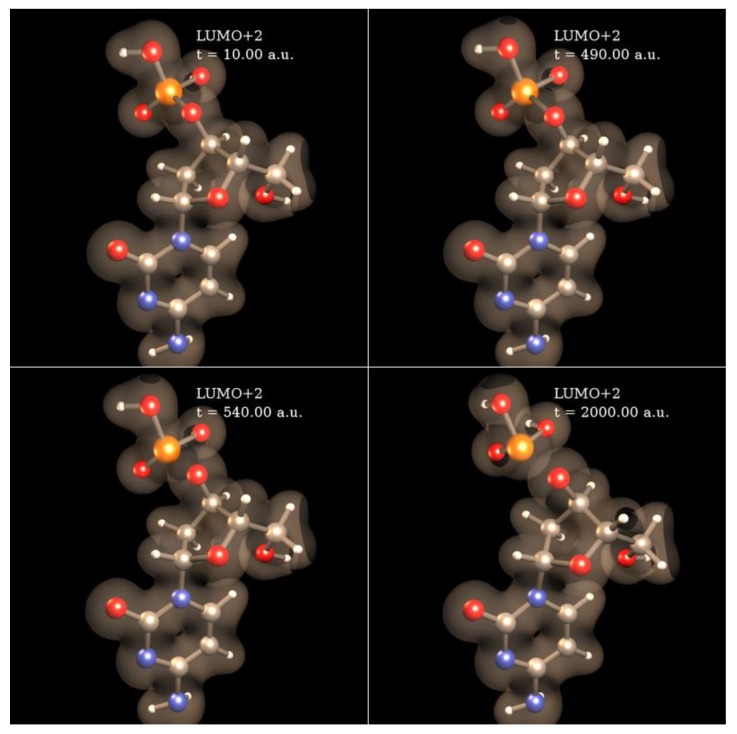
SLEND simulation of a cytosine nucleotide SSB with an electron capture at LUMO + 2$\sigma^{*}$on the phosphate. Simulation time is in a.u. At initial times (first two frames), the P–O bond along the phosphate-sugar backbone monotonically elongates; at later times (last two frames), that bond finally breaks, generating dihydrogen phosphite H_2_PO_3_^−^ and the rest of the nucleotide structure.

**Figure 10:**
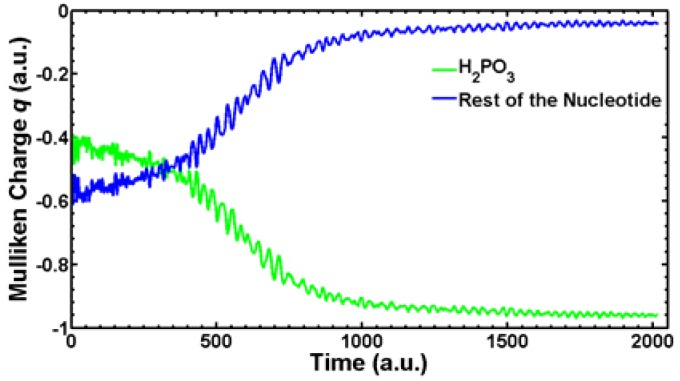
Mulliken charges vs. time of the H_2_PO_3_ moiety and the rest of the cytosine nucleotide during the SSB in Figure 9.

**Figure 11:**
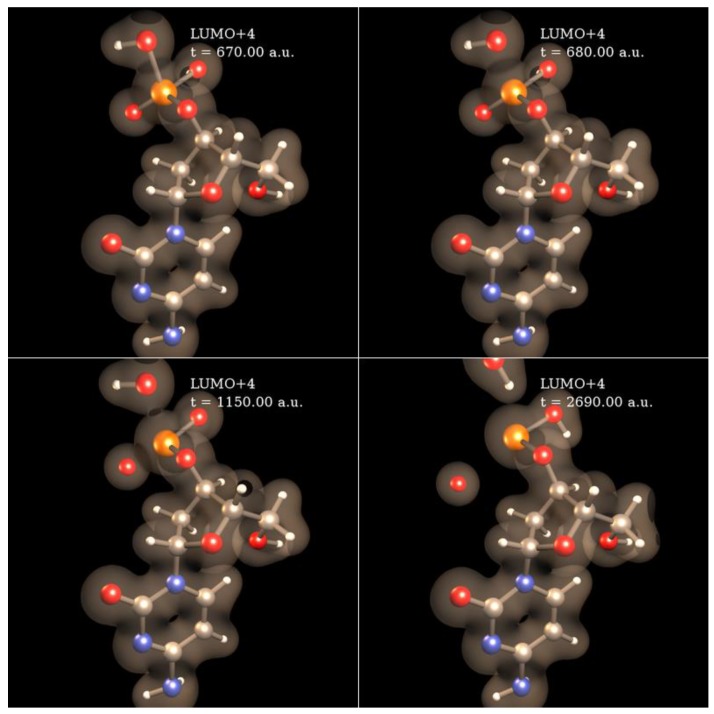
SLEND simulation of a cytosine nucleotide SSB with an electron capture at LUMO + 4$\pi^{*}$on the phosphate. Simulation time is in a.u. The P–O bond toward an OH moiety of the phosphate elongates (first panel) and finally breaks, generating an OH radical (second panel). Subsequently, the O atom of the phosphate dissociates as an O radical (third and four panels).

**Figure 12:**
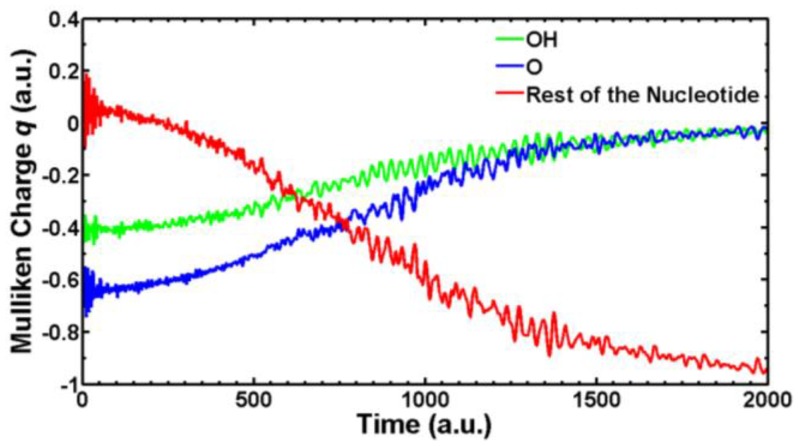
Mulliken charges vs. time of the OH and O moieties and the rest of the cytosine nucleotide during the SSB in Figure 11.

**Figure 13:**
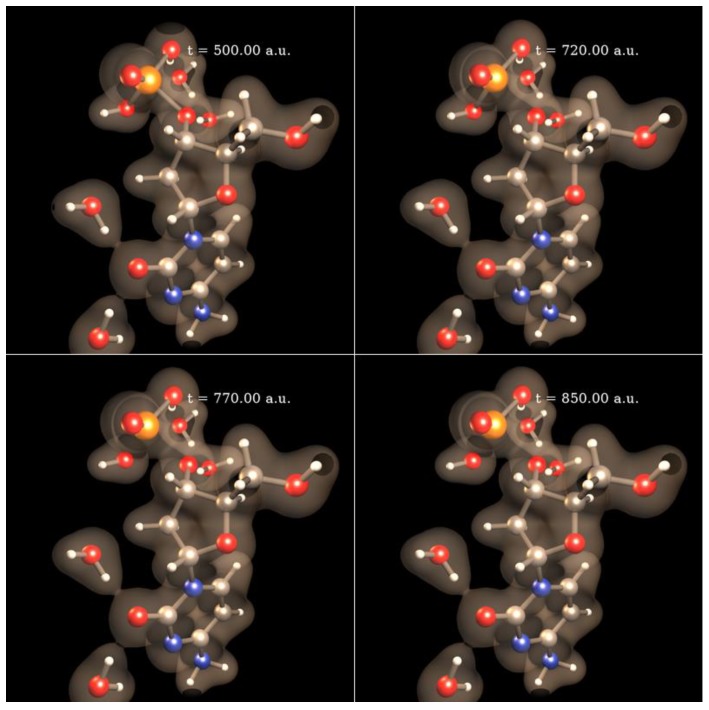
SLEND simulation of a cytosine nucleotide + 4 H_2_O with an electron capture at LUMO + 4$\pi^{*}$ on the phosphate. Simulation time is in a.u. The P–O bond along the phosphate-sugar backbone first elongates (first frame) and finally breaks, generating H_2_PO_3_ and the rest of the nucleotide structure (second frame). H_2_PO_3_ subsequently breaks into HPO_2_ and OH radicals (third and fourth frames).

**Figure 14:**
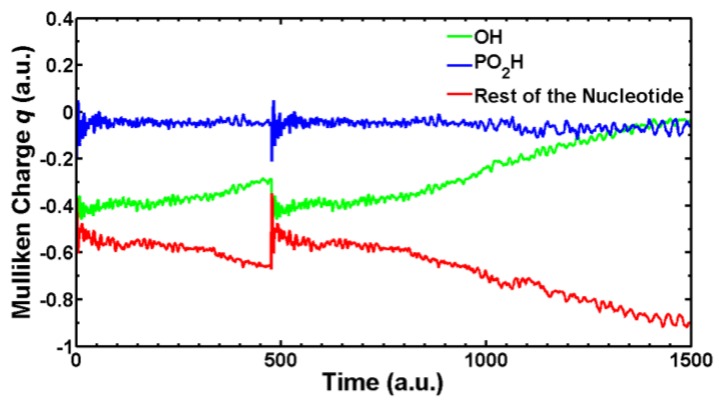
Mulliken charges vs. time of the OH and PO_2_H moieties and the rest of the cytosine nucleotide during the SSB in Figure 13.

We are currently simulating with SLEND all the types of electron-induced DNA damage discussed above. Herein, we present some preliminary results of electron-induced DNA damage in the excised cytosine nucleotide with an electron capture at the phosphate; this is the predominant type of DNA damage according to a previously discussed experiment [59]. Due to the large size of this system (161 electrons and 30 atomic centers), the STO-3G basis set is used. In the dry nucleotide with the aforesaid basis set, the first unoccupied orbitals on the phosphate are LUMO + 2$\sigma^{*}$, LUMO + 3$\pi^{*}$ and LUMO + 4$\pi^{*}$, whose topologies are shown in Figure 8. Figure 9 shows four sequential frames of a SLEND simulation of an excised cytosine nucleotide with an electron capture at LUMO + 2$\sigma^{*}$; at initial times (first two frames), the P−O bond along the phosphate-sugar backbone monotonically elongates; at later times (last two frames), that bond finally breaks, generating dihydrogen phosphite H_2_PO_3_^−^ and the rest of the nucleotide. H_2_PO_3_^−^ fragments were detected in an electron-induced DNA SSB experiment on the dibutyl phosphate ester, (C_4_H_9_)_2_HPO_4_, DNA prototype [62]. The net process in Figure 10 is a SSB involving the 3′ C−O−P phospho-ester bond. Figure 10 records the electron rearrangements during that SSB by plotting the Mulliken charges of the H_2_PO_3_ moiety and the rest of the nucleotide vs. time. H_2_PO_3_ starts with a charge of −0.6 a.u. that increases in negative value as time elapses, and reaches a value of almost −1.0 a.u. at dissociation from the nucleotide. The Mulliken charge of the rest of the nucleotide evolves to a final value of almost 0 a.u.; the total charge of the system remains equal to −1 at all times. Figure 11 shows four sequential frames of a SLEND simulation of an excised cytosine nucleotide with an electron capture at LUMO + 4$\pi^{*}$. Unlike the previous simulation, the phosphate moiety remains attached to the rest of the nucleotide but the O and OH moieties on that phosphate detach from the rest of the structure. One of the OH in this excised nucleotide is an H-capped O terminus of a cut 5′ C−O phospho-ester bond; therefore, the net process in Figure 10 can be interpreted as a SSB involving the 5′ phospho-ester bond. Figure 12 records the electron rearrangements during this SSB by plotting the Mulliken charges of the C and OH moieties and the rest of the nucleotide vs. time. Figure 13 shows four sequential frames of a SLEND simulation of the excised cytosine nucleotide hydrated with four H_2_O molecules and with an electron capture at LUMO + 4$\pi^{*}$; water is added in this case to assess solvation effects. Notice that as Figure 8 shows, solvation somewhat delocalizes the LUMO + 4$\pi^{*}$orbital over the nucleotide in contrast to the more localized orbitals of the dry sample. Nevertheless, the Mulliken population of the added electron is the highest on the phosphate. Figure 13 shows that the P–O bond along the phosphate-sugar backbone and the P–O bond toward the left OH group on H_2_PO_4_ elongate simultaneously (first frame); the P–O bond along the phosphate-sugar backbone elongates much faster than the other and breaks abruptly (first frame) into H_2_PO_3_ and the rest of the nucleotide that can be seen well separated in the second frame. The P–O bond toward the left OH group of the now detached H_2_PO_3_ continues elongating and finally breaks into HPO_2_ and OH radicals (third and fourth frames). The net process in Figure 13 is again a SSB involving the 3′ C−O−P phospho-ester bond. Finally, Figure 14 records the electron rearrangements during this SSB by plotting the Mulliken charges of the OH and PO_2_H moieties and the rest of the cytosine nucleotide vs. time. At initial times, the nearly zero charge of PO_2_H remains essentially unchanged while the charges of OH and the rest of the nucleotide gradually change as a result of the simultaneous elongations of the two P–O bonds discussed in Figure 13. At about 470 a.u. of time, a sudden rearrangement of charges among the moieties manifests as conspicuous peaks in the curves; this rearrangement corresponds to the previously mentioned abrupt break of the P–O bond along the phosphate-sugar backbone (cf. first frame of Figure 13). After this event, the charges of OH and PO_2_H evolve gradually to their final values as the H_2_PO_3_ and the rest of the nucleotide separate further and the H_2_PO_3_ breaks into PO_2_H and OH. Throughout the whole process, the nearly zero charge of HPO_2_ remains essentially unchanged although a charge transfer occurs from OH to the rest of the nucleotide through the HPO_2_ acting as a bridge.

The presented SLEND simulations of electron-induced DNA SSBs with the excised cytosine nucleotide are preliminary, and further work is necessary to complete and refine these computational studies. However, these time-dependent and non-adiabatic simulations of electron-induced DNA SSBs are the first of this type ever performed. These simulations clearly demonstrate the power of SLEND to simulate this type of process. Therefore, we are currently conducting further SLEND research on electron-induced DNA SSBs to verify and expand the discussed theoretical [26,27,28] and experimental [59,62] findings about this process.

## 4. Materials and Methods

The employed computational method, SLEND [13,31,32], adopts a total trial wavefunction $|\Psi_{Total}^{SLEND}\rangle$ = $|\Psi_{N}^{SLEND}\rangle$$|\Psi_{e}^{SLEND}\rangle$, where $|\Psi_{N}^{SLEND}\rangle$ and $|\Psi_{e}^{SLEND}\rangle$ are nuclear and electronic wavefunctions. $|\Psi_{N}^{SLEND}\rangle$ for a system having *N_N_* nuclei is the product of 3*N_N_* frozen, narrow, Gaussian wave packets:

(1) $$\langle X|\Psi_{N}^{SLEND}\left(t\right)\rangle =\langle X|R\left(t\right),\;P\left(t\right)\rangle =\prod_{A=1}^{3N_{N}}\exp\left\{-{\left[\frac{X_{A}-R_{A}\left(t\right)}{2\Delta R_{A}}\right]}^{2}+iP_{A}\left(t\right)\left[X_{A}-R_{A}\left(t\right)\right]\right\}$$

with average positions $R_{A}\left(t\right)$, average momenta $P_{A}\left(t\right)$ and widths $\left\{\Delta R_{A}\right\}$. To accelerate calculations, SLEND adopts the zero-width limit for all the nuclear wave packets: $\Delta R_{A}$ → 0 ∀A, prior to obtaining the SLEND dynamical equations (cf. Equation (3)). This generates a classical nuclear dynamics that is appropriate for describing the fast moving nuclei in PCT reactions. $|\Psi_{e}^{SLEND}\rangle$ for a system having $N_{e}$ electrons is a complex-valued, spin-unrestricted, single-determinantal wavefunction in the Thouless representation [63]:

(2) $$\begin{array}{c} \langle x|\Psi_{e}^{SLEND}\left(t\right)\rangle =\langle x|z\left(t\right),\;R\left(t\right),P\left(t\right)\rangle =\det\left\{\chi_{h}\left[x_{h};\;z\left(t\right),R\left(t\right),P\left(t\right)\right]\right\};\\ \chi_{h}=\phi_{h}+\sum_{p=N_{e}+1}^{K}z_{ph}\phi_{p}\;;\;1\leq h\leq N_{e} \end{array}$$

where $K>N_{e}$ is the size of the electronic basis set and $\left\{\chi_{h}\right\}$ are the non-orthogonal dynamical spin-orbitals (DSOs) [31]. The DSOs are linear combinations of conventional orthogonal molecular spin-orbitals (MSOs) $\left\{\phi_{h},\;\phi_{p}\right\}$ with complex-valued coefficients z(t)=$\left\{z_{ph}\left(t\right)\right\}$; the MSOs are classified as occupied $\left\{\phi_{h}\right\}$ or unoccupied $\left\{\phi_{p}\right\}$ with respect to a reference single-determinantal state |0〉=$|\phi_{1}\ldots \;\phi_{i}\ldots \;\phi_{N_{e}}\rangle$. The MSOs are constructed at initial time via a regular self-consistent-field unrestricted Hartree-Fock procedure involving K travelling electronic atomic basis functions placed on the nuclear centers. SLEND adopts the less conventional Thouless single-determinantal wavefunction [63] in Equation (2) because this prevents numerical instabilities in the SLEND dynamical equations (cf. Refs. [13,31] for full details).

The SLEND dynamical equations are obtained via the time-dependent variational principle [64] applied to the trial wavefunction $|\Psi_{Total}^{SLEND}\rangle$ [13,31]. This involves constructing the quantum Lagrangian $L_{SLEND}$ = $\langle \Psi_{Total}^{SLEND}|$i∂/∂t − $\hat{H}$$|\Psi_{Total}^{SLEND}\rangle$/$\langle \Psi_{Total}^{SLEND}|$$\Psi_{Total}^{SLEND}\rangle$, applying the zero-width limit to all the nuclear wave packets and imposing the stationary condition to the quantum action $A_{SLEND}$:$\delta A_{SLEND}$ = $\delta \int_{t_{1}}^{t_{2}}L_{SLEND}(t)dt$ =0. The described procedure generates the SLEND dynamical equations as a set of Euler-Lagrange equations: $d\left(\partial L_{SLEND}/\partial {\dot{q}}_{i}\right)/dt$ = $\partial L_{SLEND}/\partial q_{i}$, for the SLEND variational parameters {*q_i_*(*t*)}= {*R_A_*(*t*), *P_A_*(*t*), *z_ph_*(*t*), z_ph_^*^(*t*)}. The resulting SLEND dynamical equations are

(3) $$\left[\begin{array}{cccc} iC & 0 & iC_{R} & iC_{P}\\ 0 & -iC^{*} & -iC_{R}^{*} & -iC_{P}^{*}\\ iC_{R}^{†} & -iC_{R}^{T} & C_{RR} & -I+C_{RP}\\ iC_{P}^{†} & -iC_{P}^{T} & I+C_{PR} & C_{RP} \end{array}\right]\left[\begin{array}{c} \frac{dz}{dt}\\ \frac{dz^{*}}{dt}\\ \frac{dR}{dt}\\ \frac{dP}{dt} \end{array}\right]=\left[\begin{array}{c} \frac{\partial E_{Total}}{\partial z^{*}}\\ \frac{\partial E_{Total}}{\partial z^{}}\\ \frac{\partial E_{Total}}{\partial R}\\ \frac{\partial E_{Total}}{\partial P} \end{array}\right]$$

where $E_{Total}$ is the total (nuclear and electronic) energy, and the generalized non-adiabatic coupling terms are [13,31,65]

(4) (CXY)ik, jl=−2Im∂2lnS∂Xik'∂Yjl|R′=RP'=P; (CXik)ph=∂2lnS∂zph*∂Xik|R′=RP'=P;Cph, qg=∂2lnS∂zph*∂zqg|R′=RP'=P

where $X_{ik}$ and $Y_{jl}$ denote either $R_{A=i,k}$ or $P_{A=j,l}$ and S= $\langle z\left(t\right),\;R^{\prime }\left(t\right),P^{\prime }\left(t\right)|$z(t), R(t), P(t)〉. To simulate a chemical reaction, Equation (3) is integrated in time from the reactant to the product stages at initial and final times. The SLEND/KSDFT equations [13,34] are obtained by recasting all the terms in Equations (3) and (4) in a time-dependent KSDFT fashion [33].

The present SLEND and SLEND/KSDFT simulations were conducted with our own END program PACE (Python-Accelerated Coherent states Electron-nuclear dynamics, T. V. Grimes, E. S. Teixeira and J. A. Morales, Texas Tech University, 2010–2018) [13]. PACE incorporates various advanced techniques of computer science such as a mixed programming language (Python for logic flow and Fortran and C++ for numerical calculations), intra- and internode parallelization, and the fast OED/ERD atomic integral package [66] from the ACES III/IV [67] program.

## 5. Conclusions

We have presented a review of our previous [7,13,14,30] and new computational research on fundamental PCT reactions conducted with the END theory [13,31,32]. This review illustrates the capability of two END methods, SLEND and SLEND/KSDFT [13,31,32,34], to simulate PCT processes at the molecular level. The presented computational studies concentrated on three types of PCT reactions: water radiolysis reactions, proton-induced DNA damage and electron-induced DNA damage, performed on the computationally feasible prototypes: H^+^ + water clusters, H^+^ + DNA/RNA bases or + cytosine nucleotide, and electron + cytosine nucleotide, respectively. The previous SLEND study of H^+^ + (H_2_O)_1–6_ at a Bragg-peak energy of 100 keV [14] attempted to progressively capture bulk water radiolysis processes through a series of 10 water clusters culminating in the prism (H_2_O)_6_ isomer; the latter is considered the “smallest drop of water” [40,41]. The H^+^ + H_2_O simulations at 100 keV provide cluster-to-proton bound-state-to-bound-state one-electron-transfer total integral cross sections in satisfactory agreement with available experimental [46,47,48,49] and theoretical results [25,37]. Furthermore, the H^+^ + (H_2_O)_2–6_ simulations at 100 keV provide the same type of integral cross sections for clusters beyond the H_2_O monomer for the first time; these unique data call for further corroboration by future experiments and calculations on the same systems. In addition, another SLEND study of H^+^ + (H_2_O)_2–3_ at 1 keV [7], an energy representative of post-Bragg-peak and/or secondary H^+^s, illustrates the capability of SLEND to predict the formation of DNA-damaging species such as H, O and OH radicals. The previous SLEND and SLEND/KSDFT study of proton-induced DNA damage with H^+^ + DNA/RNA bases at a Bragg-peak energy of 80 keV provides base-to-proton bound-state-to-bound-state one-electron-transfer total integral cross sections in satisfactory agreement with available theoretical results [24,50]. However, the four types of theoretical results (the SLEND and SLEND/KSDFT ones and three alternative ones [24,50]) are lower in value than the only available experimental results [51]. The reason for this discrepancy is unknown, but it is notable that four theoretical methods highly differing in nature predict similar integral cross sections. The new SLEND studies concentrate on proton-induced DNA damage reactions performed on the H^+^ + cytosine nucleotide prototype and electron-induced DNA damage performed on the electron + cytosine nucleotide prototype. To the best of our knowledge, these are the first time-dependent and non-adiabatic simulations performed on these large systems. These simulations reveal various fragmentation reactions occurring as the impact sites of the H^+^s or the capturing molecular orbitals of the electrons vary. These simulations mark an important milestone in the END research on PCT reactions due to the challenging size of the nucleotide and the complexity of the described processes (e.g., the novel treatment of electron-induced DNA damage within the END framework).

The presented studies predicted some relevant cross sections and elucidated some microscopic details of three types of PCT reactions. While these results are significant, further research is necessary to turn END into a highly predictive method for PCT reactions. For instance, to improve the END accuracy for PCT, it is necessary to conduct predictions of additional dynamical properties, compare them with alterative experimental and theoretical data, and modify the END framework in case of disagreement. To improve the END relevance for PCT, it is necessary to utilize larger prototypes that get closer to real-life systems (e.g., water radiolysis reactions should be subsequently simulated with water clusters larger than (H_2_O)_6,_ and proton-induced and electron-induced DNA damage reactions should be subsequently simulated with systems containing more than one nucleotide unit and a larger number of H_2_O molecules for solvation). The presented methods and results provide a firm base to undertake all those improvements that are currently underway in our research group.

## 6. Recognition

Upon completing this manuscript in its final form, a very recent study of H^+^ + (H_2_O)*_n_*, *n* = 2–4, 6, 10 and 20, with the screened independent atom model (SIAM) came to our attention [68]. The authors of Ref. [68] found that the cluster-to-proton bound-state-to-bound-state one-electron-transfer total integral cross sections, $\sigma_{1-ET}$, of H^+^ + (H_2_O)*_n_* scales according to our derived formula $\sigma_{1-ET}\left(n\right)=cn^{2/3}$ (cf. Section 3.1 and our Ref. [14]) at a collisional energy = 10 keV but not at our studied energy = 100 keV; at that energy, the authors of [68] found a nearly linear scaling, $\sigma_{1-ET}\left(n\right)=cn$. There are no available experimental or alternative theoretical data to resolve this discrepancy. Furthermore, the two employed methods differ substantially in their structures (SLEND is an ab initio method to describe various chemical processes including fragmentations and SIAM is a model focused on electron captures and ionization processes) and incorporate different approximations. Due to all these complicating factors, we cannot currently speculate about the origin of the present discrepancy. We expect that future calculations and experiments will shed light on this result.
